# Investigation of HLA susceptibility alleles and genotypes with hematological disease among Chinese Han population

**DOI:** 10.1371/journal.pone.0281698

**Published:** 2024-04-09

**Authors:** Ye-Mo Li, Yu-Xia Li, Dai-Yang Li, Ying Zhou, Lin An, Zhi-Yang Yuan, Ke-Ming Du, Zhong-Zheng Zheng

**Affiliations:** 1 Shenzhen Tissuebank Precision Medicine Co., Ltd, Shenzhen, China; 2 Shanghai Tissuebank Biotechnology Co., Ltd, Shanghai, China; National and Kapodistrian University of Athens, GREECE

## Abstract

Several genes involved in the pathogenesis have been identified, with the human leukocyte antigen (HLA) system playing an essential role. However, the relationship between HLA and a cluster of hematological diseases has received little attention in China. Blood samples (n = 123913) from 43568 patients and 80345 individuals without known pathology were genotyped for HLA class I and II using sequencing-based typing. We discovered that HLA-A*11:01, B*40:01, C*01:02, DQB1*03:01, and DRB1*09:01 were prevalent in China. Furthermore, three high-frequency alleles (DQB1*03:01, DQB1*06:02, and DRB1*15:01) were found to be hazardous in malignant hematologic diseases when compared to controls. In addition, for benign hematologic disorders, 7 high-frequency risk alleles (A*01:01, B*46:01, C*01:02, DQB1*03:03, DQB1*05:02, DRB1*09:01, and DRB1*14:54) and 8 high-frequency susceptible genotypes (A*11:01-A*11:01, B*46:01-B*58:01, B*46:01-B*46:01, C*01:02-C*03:04, DQB1*03:01-DQB1*05:02, DQB1*03:03-DQB1*06:01, DRB1*09:01-DRB1*15:01, and DRB1*14:54-DRB1*15:01) were observed. To summarize, our findings indicate the association between HLA alleles/genotypes and a variety of hematological disorders, which is critical for disease surveillance.

## Introduction

Hematological disorders are becoming a global public health problem, which comprise a large number of malignant and benign hematologic manifestations, including leukocyte abnormalities, erythrocyte disorders, hemorrhagic disease, and myeloproliferative disorders. It is well-known that blood diseases often lead to shortened lifespans and poor quality of life. With the development of gene therapy, previous studies have confirmed that genetic factors influence the treatment of blood disorders [1]. As a result, the research of disease-susceptibility genes might promote the clinical formulation of reasonable treatment plans.

The human leukocyte antigen (HLA) gene complex is responsible for encoding the HLA, commonly known as the major histocompatibility complex (MHC) in humans. The HLA gene occupies a 7.6 Mb region on the short arm of chromosome 6 (6p21.31), which is home to more than 250 genes with diverse roles and is currently thought to have the highest gene density and the most polymorphisms of any human chromosomal region [2]. Based on their prevalence and role, HLA antigens are classified into three classes: classes I, II, and III. HLA class I antigens include HLA-A, -B, and -C; HLA class II antigens include HLA-DP, -DQ, and -DR; nonclassical HLA class I and II molecules include HLA-F, -E, -H, -X, -DN, -DO, and -DM; and others, like complement, are class III antigens [3, 4]. HLA is highly polymorphic, and alleles/genotypes have been reported to be associated with over 500 diseases [5–8]. Research into blood system diseases (autoimmune diseases accounting for a large proportion), tumor immunity, and transplant immunity all benefit greatly from the exploration of HLA, as it is directly related to the immune response, regulation, and monitoring of the body [9]. More importantly, the HLA gene plays a pivotal role in successful hematopoietic stem cell transplantation (HSCT) [10], including but not limited to unrelated HLA-matched donor searches, donor registry planning, population genetic studies, and anthropogenesis studies [11]. What’s more, HLA polymorphism has been shown to be associated with prognosis in hematological patients [12]. Studies have revealed that certain alleles are indeed associated with the occurrence of different types of hematological diseases, such as the comparison of clinical outcomes between HLA allele matched and 1–2 alleles mismatched unrelated allogeneic HSCT [13], the association between HLA-A, -B, and -DRB1 alleles and Chinese Han leukemia [14], and the correlation between HLA class I and II genes and susceptibility to aplastic anemia (AA) [15–17]. However, the majority of them are constrained by low-resolution genotyping data and research scale. Undoubtedly, due to the distribution of alleles/genotypes in the HLA system varying between different populations [11], it is critical to observe independent evidence on the association between HLA and hematological diseases in China, which contributes significantly to a better understanding of the immunological mechanisms involved in the disease.

In the present study, we targeted to compare the alleles and genotypes at five HLA loci (-A, -B, -C, -DRB1, and -DQB1) **between** hematologic patients and individuals without known pathology in China. Importantly, we paid close attention to the association of HLA class I and II with malignant and benign hematologic diseases, followed by the distribution of HLA alleles/genotypes and the presentation of how disease-associated genes are defined.

## Materials and methods

### Study population

From June 2012 to July 2021, blood samples were collected from Han population from over 600 hospitals in 29 provinces across China. All subjects received written informed consent for the study. This study was approved by the Ethics Committee of Shanghai Tissuebank Medical Laboratory.

Herein, the malignant hematologic diseases included acute lymphatic leukemia (ALL), chronic myeloid leukemia (CML), acute myeloid leukemia (AML), myelodysplastic syndromes (MDS), and lymphoma. The benign hematologic diseases in this study were composed of hemophagocytic lymphohistiocytosis (HLH) occurring post allogeneic HSCT for benign disorders without involving malignancy-associated HLH, AA, and thalassemia. Leukemia diagnosis followed the French-American-British (FAB) classification. Diagnosis of other disease types was according to previously reported criteria.

**Fig 1:**
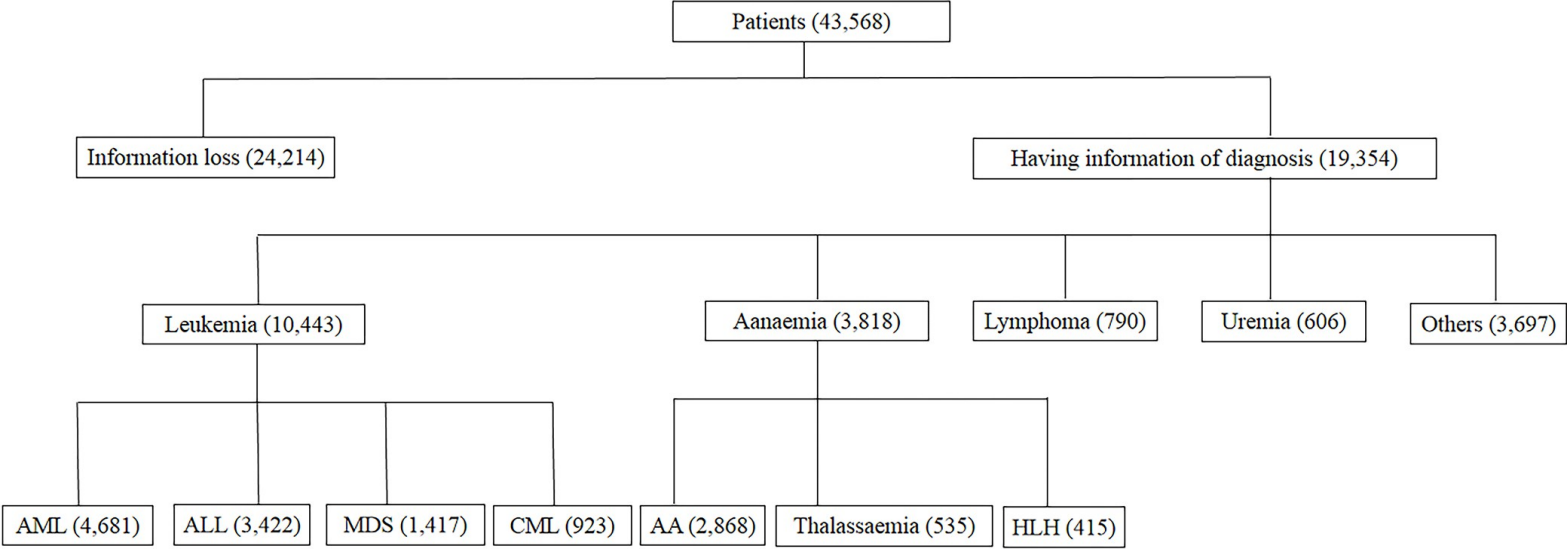
Description of disease classification of the patients in this study.

A total of 123913 blood samples, including 43568 from patients and 80345 from individuals without known pathology, were analyzed in this retrospective study. There were 19354 patients with precise diagnostic information, of whom 10443 had been diagnosed with malignant hematologic diseases (3422 of ALL, 4681 of AML, 923 of CML, and 1417 of MDS). Patients with benign hematologic diseases consisted of 415 with HLH, 2868 with AA, and 535 with thalassemia. There were 790 cases of lymphoma and 4303 cases of other diseases (Fig 1).

### DNA extraction and HLA typing

Blood samples were collected from patients and controls. Genomic DNA was extracted from blood using the QIARamp Blood Kit (Qiagen, Hilden, Germany). HLA class I (-A, -B, and -C) and II (mainly, -DRB1 and -DQB1) alleles were subtyped based on the IMGT/HLA Database release 3.16 (http://www.ebi.ac.uk/ipd/imgt/hla/stats.html) as reported before [11], using primer pairs recommended by the International Histocompatibility Working Group (IHWG) [18]. High-resolution genotyping of HLA groups was performed using the sequencing-based typing (SBT) method by the HLA Genotyping Kit (Shanghai Tissuebank Biotech, Shanghai, China). The reaction conditions were as follows: pre-denaturation at 96°C for 2 min, denaturation at 96°C for 15 s, annealing at 65°C for 15 s, extension at 72°C for 1 min, 40 cycles, extension at 72°C for 7 min, and storage at 4°C. The reaction product was stored at 4°C. 5 μl of the amplification product was extracted by 1.5% agarose gel electrophoresis and the results were observed under ultraviolet light.

### Statistical analysis

Allele frequency was calculated by referring to the Chinese common and well-documented (CWD) HLA Allele List [19] as follows: allele frequency = number of genes/(total number of a disease*2), genotype frequency = number of genotypes/total number of diseases. HLA allele frequency and genotype were determined by the direct counting method.

All analyses were performed using R version 4.1.0. A Chi-square test was applied to compare the variability of HLA alleles and genotypes between patients and individuals without known pathology. The prevalence of patients was counted according to diagnostic information, and the association strength was estimated by odds ratio (OR) and 95% confidence interval (CI). Then, Bonferroni’s correction was used to adjust the P-value, which was considered statistically significant when it was less than 0.05.

## Results

### Characteristics of HLA alleles frequency

**Fig 2:**
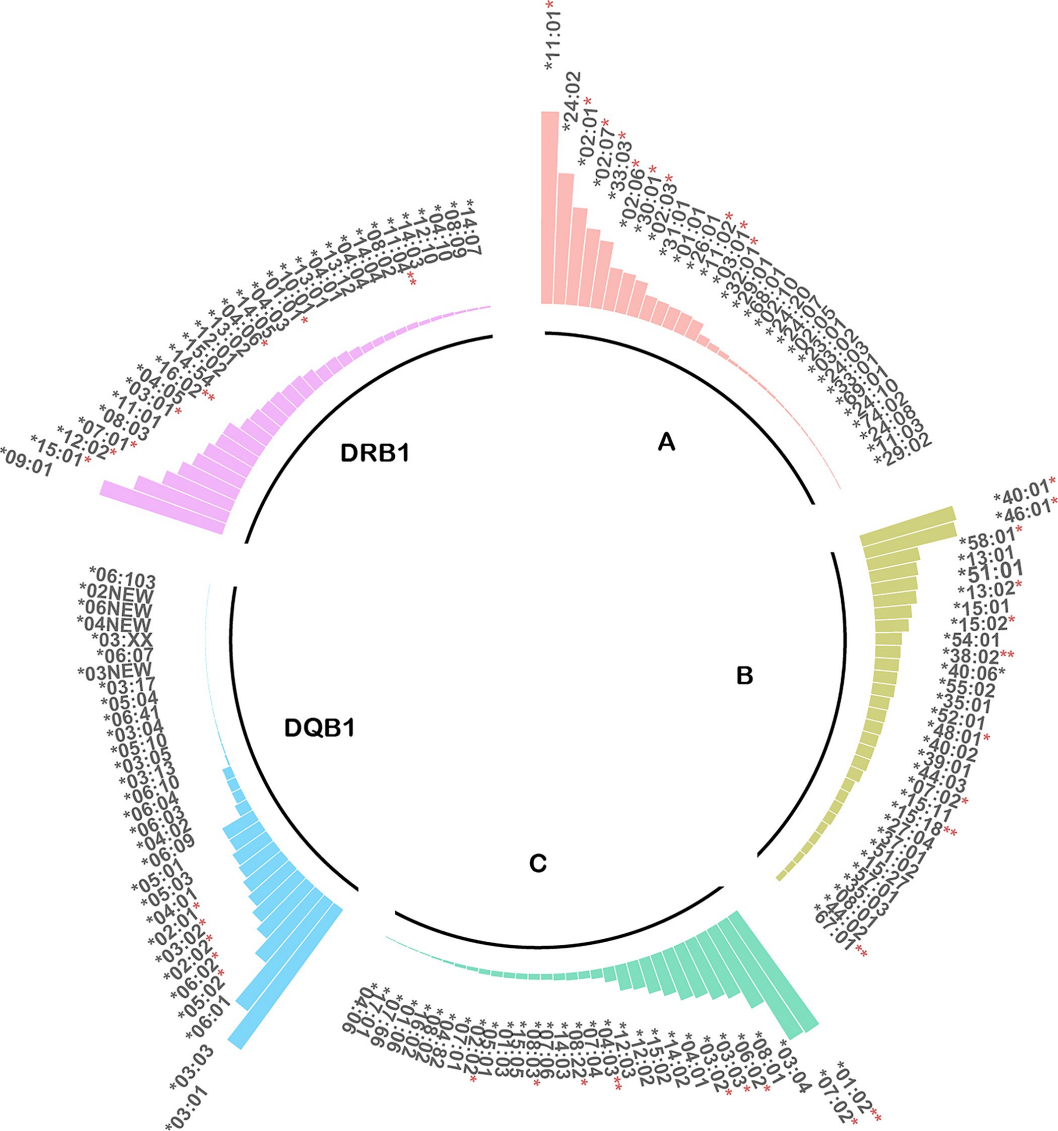
Characteristics of HLA alleles frequency. Frequencies of HLA-A, -B, -C, -DQB1, -DRB1 alleles between patients and control subjects were compared. *Pc <0.05, **Pc <0.01.

In total, 202 alleles of HLA-A, 282 of HLA-B, 173 of HLA-C, 86 of HLA-DQB1 and 149 of DRB1 were analyzed. Alleles A*11:01 (22.91%), A*24:02 (15.61%), A*02:01 (11.63%), A*02:07 (9.30%), and A*33:03 (8.08%) had the highest frequencies at HLA-A locus. B*40:01 (11.62%), B*46:01 (11.22%), B*58:01 (6.31%), B*13:01 (5.74%), and B*51:01 (5.43%) were the top five high-frequency HLA-B alleles. Details and the high-frequency alleles of HLA-C, -DQB1, and -DRB1 are shown in Fig 2. Among them, A*11:01, A*02:01, A*02:07, A*33:03, B*40:01, B*46:01, B*58:01, C*01:02, C*07:02, C*06:02, DQB1*05:02, DQB1*06:02, DRB1*15:01, DRB1*12:02, and DRB1*07:01 had significantly differences between patients and controls (Pc <0.05).

### Potential protective and risk HLA-A, -B, -C, -DQB1, and -DRB1 alleles and genotypes

#### Malignant hematologic diseases

As seen in Table 1, there were four protective and two risk alleles in AML: the former included A*11:01 (OR: 0.91; 95% CI: 0.86–0.95, Pc = 0.02), B*13:11 (OR: 0.85; 95% CI: 0.77–0.93, Pc = 0.04), C*03:04 (OR: 0.85; 95% CI: 0.79–0.92, Pc = 0.02), and DQB1*05:02 (OR: 0.82; 95% CI: 0.76–0.89, Pc = 0.02), while the latter included A*03:01 (OR: 1.30; 95% CI: 1.15–1.48, Pc = 0.02) and DQB1*03:01 (OR: 1.09; 95% CI: 1.04–1.15, Pc = 0.05). S1 Table displayed the HLA alleles with significant differences at each locus in AML patients compared to controls (excluding the highest-frequency alleles at each locus).

**Table 1:** Differences in the most common HLA-A, -B, -C, -DQ, and -DR alleles between patients with malignant hematologic disorders and controls.

Disease	HLA allele	Frequency in patients (%)	Frequency in controls (%)	OR^a^ (95%CI)	P	Pc^b^
**AML**	A*11:01	21.67	23.39	0.91 (0.86–0.95)	<0.01	0.02
	B*13:01	4.92	5.76	0.91 (0.86–0.95)	<0.01	0.04
	C*03:04	9.45	10.91	0.85 (0.77–0.93)	<0.01	0.02
	DQB1*03:01	22.17	20.74	1.09 (1.04–1.15)	<0.01	0.05
**MDS**	A*11:01	19.37	23.39	0.79 (0.72–0.86)	<0.01	0.01
	B*46:01	8.05	11.45	0.68 (0.59–0.78)	<0.01	0.01
	C*01:02	14.68	16.84	0.85 (0.77–0.94)	<0.01	0.02
	DQB1*06:02	10.23	7.08	1.50 (1.32–1.69)	<0.01	0.01
	DRB1*15:01	13.62	11.53	1.21 (1.09–1.35)	<0.01	0.01

^a^OR, odds ratio, which describes the odds of cases being HLA alleles carriers to the odds of controls being HLA alleles carriers. Specifically, OR = 1 indicates that the factor has no impact on the occurrence of the disease, OR >1 indicates that the factor is a risk factor, and OR <1 indicates that the factor is protective.

^b^Pc values for multiple comparisons were corrected by Bonferroni’s correction.

In contrast, MDS seems to have more protective and risk factors for this study, corresponding to 11 (including 3 of HLA-A, 2 of HLA-B, 3 of HLA-C, 2 of HLA-DRB1, and 1 of HLA-DQB1) and 14 (including 5 of HLA-B, 3 of HLA-C, 3 of HLA-A, 2 of HLA-DRB1, and 1 of HLA-DQB1) alleles. For high-frequency alleles, there were A*11:01 (OR: 0.79; 95% CI: 0.72–0.86, Pc = 0.01), B*46:01 (OR: 0.68; 95% CI: 0.59–0.78, Pc = 0.01), C*01:02 (OR: 0.85; 95% CI: 0.77–0.94, Pc = 0.02). and DQB1*06:02 (OR: 1.50; 95% CI: 1.32–1.69, Pc = 0.01). Except for the high-frequency alleles at each locus, the other HLA alleles with significant differences at each locus in MDS patients compared to controls were shown in S2 Table.

#### Benign hematologic diseases

As shown in Table 2, it is clear that anemia had the largest number of alleles with potential protective and risk effects in the present study. Among them, HLH possessed 3 protective alleles [i.e., B*15:02 (OR: 0.43; 95% CI: 0.26–0.72), Pc = 0.02; C*08:01 (OR: 0.61; 95% CI: 0.45–0.83, Pc = 0.02); and DQB1*03:01 (OR: 0.74; 95% CI: 0.62–0.89, Pc = 0.04)] and 2 risk alleles [i.e., A*01:01 (OR: 2.04; 95% CI: 1.50–2.76, Pc = 0.02) and DRB1*10:01 (OR: 2.54; 95% CI: 1.76–3.66, Pc = 0.02)]. Twenty-three alleles expressed protective effects against thalassemia, most of which were at loci A and B (6 and 8, respectively), and the remaining at loci C, DRB1 and DQB1 (3 of each), among which A*02:01 (OR: 0.40; 95% CI: 0.30–0.53, Pc <0.01) and C*03:03 (OR: 0.62; 95% CI: 0.46–0.84, Pc = 0.01) had the highest frequencies. While B*46:01 (OR: 1.67; 95% CI: 1.43–1.96, Pc <0.01), DQB1*05:02 (OR: 3.05; 95% CI: 2.64–3.52, Pc <0.01), and DRB1*14:54 (OR: 2.76; 95% CI: 2.24–3.40, Pc <0.01) were the highest frequency among 20 risk alleles (A/B/C/DRB1/DQB1 loci correspond to 3, 5, 5, 4, and 3 alleles, respectively).

**Table 2:** Differences in the most common HLA-A, -B, -C, -DQ, and -DR alleles between patients with benign hematologic disorders and controls.

Disease	HLA allele	Frequency in patients (%)	Frequency in controls (%)	OR^a^ (95%CI)	P	Pc^b^
**HLH**	A*01:01	5.30	2.68	2.04 (1.50–2.76)	<0.01	0.02
	B*15:01	6.75	4.39	1.57 (1.20–2.07)	<0.01	0.04
	C*08:01	5.30	8.41	0.61 (0.45–0.83)	<0.01	0.02
	DQB1*03:01	16.27	20.74	0.74 (0.62–0.89)	<0.01	0.04
	DRB1*10:01	3.61	1.46	2.54 (1.76–3.66)	<0.01	0.02
**AA**	A*11:01	21.81	23.39	0.91 (0.86–0.97)	0.01	0.03
	B*40:01	9.59	11.91	0.78 (0.72–0.86)	<0.01	<0.01
	C*07:02	14.54	16.59	0.86 (0.79–0.92)	<0.01	<0.01
	DQB1*03:03	22.82	16.42	1.51 (1.41–1.60)	<0.01	<0.01
	DRB1*09:01	22.02	15.32	1.56 (1.46–1.66)	<0.01	<0.01
**Thalassemia**	A*11:01	31.68	23.39	1.52 (1.33–1.73)	<0.01	<0.01
	B*46:01	17.76	11.45	1.67 (1.43–1.96)	<0.01	<0.01
	C*01:02	21.31	16.84	1.34 (1.15–1.55)	<0.01	<0.01
	DQB1*05:02	22.43	8.66	3.05 (2.64–3.52)	<0.01	<0.01
	DRB1*14:54	9.25	3.56	2.76 (2.24–3.40)	<0.01	<0.01

^a^OR, odds ratio, which describes the odds of cases being HLA alleles carriers to the odds of controls being HLA alleles carriers. Specifically, OR = 1 indicates that the factor has no impact on the occurrence of the disease, OR >1 indicates that the factor is a risk factor, and OR <1 indicates that the factor is protective.

^b^Pc values for multiple comparisons were corrected by Bonferroni’s correction.

The protective alleles for AA were mostly at HLA-DQB1 locus (10/32), with the highest frequency of A*11:01 (OR: 0.91; 95% CI: 0.86–0.97, Pc = 0.03), B*40:01 (OR: 0.78; 95% CI: 0.72–0.86, Pc <0.01), C*07:02 (OR: 0.86; 95% CI: 0.79–0.92, Pc <0.01), and DQB1*03:01 (OR: 0.90; 95% CI: 0.84–0.96, Pc <0.01). HLA alleles at loci B and C accounted for more than half of the risk factors for AA (8/26 and 7/26, respectively); interestingly, the top two high-frequency risk alleles all belonged to class II [i.e., DQB1*03:03 (OR: 1.51; 95% CI: 1.41–1.60, Pc <0.01) and DRB1*09:01 (OR: 1.56; 95% CI: 1.46–1.66, Pc <0.01)]. S3–S5 Tables displayed the HLA alleles with significant differences at each locus in patients with HLH, AA, and thalassemia compared to controls (excluding the highest-frequency alleles at each locus).

### Potential protective and risk HLA-A, -B, -C, -DQB1, and -DRB1 genotypes

We detected a total of 220 HLA-A-A genotypes, 198 HLA-B-B genotypes, 225 HLA-C-C genotypes, 182 HLA-DQB1-DQB1 genotypes, and 210 HLA-DRB1-DRB1 genotypes. Unfortunately, no risk/protective genotypes were found to be associated with malignant hematologic diseases. But we observed the following susceptibility and antagonistic genotypes for benign hematologic disorders.

Herein, 27 HLA genotypes seem to be protective factors for AA, including 8 of HLA-A, 7 of HLA-B, 3 of HLA-C, 3 of HLA-DQB1 and 6 of HLA-DRB1, with the more frequent ones of A*11:01-A*24:02 (OR: 0.70; 95% CI: 0.59–0.82, Pc <0.01), C*01:02-C*07:02 (OR: 0.80; 95% CI: 0.67–0.95, Pc = 0.03), and DQB1*03:01-DQB1*06:01 (OR: 0.67; 95% CI: 0.54–0.83, Pc <0.01). Whereas B*46:01-B*46:01 (OR: 1.67; 95% CI: 1.32–2.12, Pc <0.01) and DRB1*09:01-DRB1*15:01 (OR: 1.77; 95% CI: 1.51–2.08, Pc <0.01) occurred most frequently in the 25 risk genotypes for AA (Table 3).

**Table 3:** Variations in the most common HLA-A-A, B-B, C-C, DQ-DQ, and DR-DR genotypes in the benign hematopoietic patients and controls.

Disease	HLA genotype	Frequency in patients (%)	Frequency in controls (%)	OR^a^ (95%CI)	P	Pc^b^
**AA**	A*11:01-A*24:02	5.20	7.30	0.70 (0.59–0.82)	<0.01	<0.01
	B*46:01-B*46:01	2.58	1.56	1.67 (1.32–2.12)	<0.01	<0.01
	C*01:02-C*07:02	4.71	5.83	0.80 (0.67–0.95)	0.01	0.03
	DQB1*03:03-DQB1*06:01	8.12	3.49	2.44 (2.12–2.81)	<0.01	<0.01
	DRB1*09:01-DRB1*15:01	5.79	3.35	1.77 (1.51–2.08)	<0.01	<0.01
**Thalassemia**	A*11:01-A*11:01	10.84	6.03	1.89 (1.44–2.49)	<0.01	<0.01
	B*46:01-B*58:01	3.74	1.64	2.32 (1.48–3.65)	<0.01	<0.01
	C*01:02-C*03:04	7.66	3.83	2.08 (1.51–2.87)	<0.01	<0.01
	DQB1*03:01-DQB1*05:02	7.66	3.41	2.35 (1.70–3.24)	<0.01	<0.01
	DRB1*14:54-DRB1*15:01	3.18	0.84	3.87 (2.38–6.32)	<0.01	<0.01

^a^OR, odds ratio, which describes the odds of cases being HLA alleles carriers to the odds of controls being HLA alleles carriers. Specifically, OR = 1 indicates that the factor has no impact on the occurrence of the disease, OR >1 indicates that the factor is a risk factor, and OR <1 indicates that the factor is protective.

^b^Pc values for multiple comparisons were corrected by Bonferroni’s correction.

For thalassemia, a statistically significant difference in HLA genotypes between patients and controls was observed, with only one protective genotype A*02:01-A*24:02 (OR: 0.36; 95% CI: 0.17–0.76, Pc = 0.03). A total of 22 risk genotypes of thalassemia consisted of 13 class I (5 for A, 6 for B, and 2 for C) and 9 class II genotypes (5 and 4 for DQB1 and DRB1, respectively), with B*46:01-B*58:01 (OR: 2.32; 95% CI: 1.48–3.65, Pc = 0.01), C*01:02-C*03:04 (OR: 2.08; 95% CI: 1.51–2.87, Pc <0.01), DQB1*03:01-DQB1*05:02 (OR: 2.35; 95% CI: 1.70–3.24, Pc <0.01), and DRB1*14:54-DRB1*15:01 (OR: 3.87; 95% CI: 2.38–6.32, Pc <0.01) occurred most frequently (Table 3). S6 and S7 Tables exhibited the genotypes with significant differences in each locus (excluding the highest-frequency genotypes for each locus) between AA and thalassemia patients and the control group, respectively.

## Discussion

As the first large-scale HLA individual study in China, it conducted a comprehensive analysis spanning 9 years, covering various malignant and benign hematologic diseases. The highlight is the associations of HLA genes with a range of onco-hematological diseases. In this study, alleles and genotypes of HLA-A, -B, -C, -DQ, and -DR were collected from 43568 patients and 80345 individuals without known pathology. This approach has revealed over 100 disease-associated loci (28 HLA-A, 37 HLA-B, 28 HLA-C, 21 HLA-DQB1, and 24 HLA-DRB1) and has provided insights into the hematologic disease surveillance.

Nevertheless, in this study, some high-frequency alleles and genotypes were common in both patients and individuals without known pathology. We discovered that HLA-A*11:01, B*40:01, C*01:02, DQB1*03:01, and DRB1*09:01 were prevalent in China, which has been confirmed by Zhou et al [11]. Notably, three high-frequency alleles (DQB1*03:01, DQB1*06:02, DRB1*15:01) were susceptible genes of malignant hematologic diseases. For benign hematologic disorders, we observed seven high-frequency alleles (A*01:01, B*46:01, C*01:02, DQB1*03:03, DQB1*05:02, DRB1*09:01, DRB1*14:54) and eight high-frequency genotypes (A*11:01-A*11:01, B*46:01-B*58:01, B*46:01-B*46:01, C*01:02-C*03:04, DQB1*03:01-DQB1*05:02, DQB1*03:03-DQB1*06:01, DRB1*09:01-DRB1*15:01, DRB1*14:54-DRB1*15:01) were considered as risk factors. Thus, these alleles and genotypes should be paid more attention.

For malignant hematologic diseases, Wang et al. [20] found that A*11:01 and DQB1*05:02 might be protective genes of AML, consistent with our results. Qi et al. [21] reported a higher frequency of DRB1*15:01 in Chinese ALL patients compared to controls as a risk allele (OR: 1.70; 95% CI: 1.24–2.33), and higher frequency of A*02:07 (Pc = 0.013), A*29:01 (Pc = 0.044), B*07:02 (Pc = 0.029), B*07:05:01G (Pc = 0.044) and B*35:02 (Pc = 0.023), but a lower frequency of A*02:03 in AML patients compared to controls (0.79% vs 3.10%, Pc = 0.011). Also, DRB1*11:28 occurred at a significantly higher frequency in the CML group compared with the controls (Pc = 0.015). Actually, our study further expanded the HLA alleles of AML and ALL susceptibility by increasing the sample size based on previous studies. Furthermore, Xiao et al. [22] reported that the frequency of the HLA-DR15 expression in MDS patients (38.7%) was significantly higher than that in healthy controls (Pc <0.01). The existence of the HLA-DR15 allele suggested MDS susceptibility, which is consistent with our findings. Wang et al. [20] also found that B*46:01 and DQB1*06:02 may be protective and susceptible genes for MDS, respectively, consistent with the results of this study. Uçar et al. [23] discovered higher frequencies of HLA-A*29 (OR: 5.65; Pc = 0.001), B*07 (OR: 3.00; Pc = 0.003), and DRB1*11 (OR: 1.80; Pc = 0.002) alleles in patients with Hodgkin’s lymphoma compared with controls. Significantly increased allele frequencies of HLA-DQA1*01:03 and HLA-DQB1*06:01 have also been reported in lymphoma [24]. Undoubtedly, malignant hematologic diseases are mainly because of the occurrence of molecular abnormalities leading to the deregulation of immature hematopoietic cells [25]. Therefore, HLA loci are expected to be a novel biomarker for the clinical diagnosis of malignant hematologic disorders.

We sought to determine HLA-related genes for benign hematologic diseases by comparing the findings of previous studies. However, there have been no studies on the association between HLH and HLA alleles in recent years, it must be pointed out we are the most comprehensive study of the Chinese population. Although Wang et al. [20] enrolled patients with thalassemia for disease correlation analysis, no substantive conclusions have been drawn. Relatively, the association between HLA class II and autoimmune blood diseases (especially AA) has been reported more frequently. Consistent with our results, Wang et al. [20] reported that A*02:01, A*02:06, B*15:11, and DQB1*06:02 might be susceptible genes for AA, while A*33:03, B*58:01, C*03:02, DRB1*16:02, and DQB1*05:02 might be protective genes. Yari et al. [26] reported DRB1*07 at a frequency of 8.3% in normal subjects and 15.7% in AA patients. Notably, Qi et al. [15] reported HLA-DRB1*15:01 (OR: 2.11; Pc = 2.87×10^−3^) and DQB1*06:02 (OR: 2.01; Pc = 1.86×10^−2^) were risk alleles of AA in Han population from northern China, consistent with our findings. They also found HLA-DRB1*15:01-DQB1*06:02 (OR: 2.09; Pc = 4.90×10^−3^) and DRB1*14:05-DQB1*05:03 (OR: 2.82; Pc = 2.65×10^−2^) were strongly associated with AA. Therefore, it could be speculated that the HLA diversity may be responsible for the differences between these studies. In addition, in a study of thalassemia patients in Iranian, HLA-DRB1*15:03 allele frequency was significantly different between 59 alloimmunised and 205 non-alloimmunised patients (OR: 4.193; Pc = 0.0001) [27]. These preliminary data laid the foundation for further research of HLA in benign hematologic diseases. Collectively, susceptible HLA alleles and genotypes may be considered a promising aspect of clinical surveillance.

In China, there are regional distributions of several allelic categories meaning that individuals from each of the geographic regions shared certain common alleles and haplotypes with distinctive genetic characteristics. The A*02:01-C*03:03-B*15:11-DRB1*15:01-DQB1*06:02 haplotype was reported relatively common in the Northeast and North China regions [11]. In this study, DQB1*06:02, and DRB1*15:01 alleles were found to be hazardous in malignant hematologic diseases when compared to controls. This may provide useful information for selecting donors without unfavorable alleles in different regions under feasible conditions. Given that the regional distribution of allele categories may affect the analysis data, our current conclusion needs to be further confirmed in further exploration based on regions.

There are several limitations to our study. First of all, the primary limitation is the paucity of diagnostic information for some patients, but our sample size is relatively adequate in comparison to previous studies. Considering the errors caused by multiple comparisons, we used Bonferroni’s correction for adjustments. Nonetheless, it may produce excessively conservative results when the number of multiple tests exceeds 10 [28, 29]. In addition, there is no consensus regarding the function of risk alleles, such as chromosomal abnormalities and other forms of clonal evolution. Actually, some of our findings appear to contradict previous reports. This is possibly owing to deviations in cohort size, ethnic and regional distribution. Notably, since patients with malignancy-associated HLH were ruled out before analysis, the association between HLA alleles/genotypes and malignancy-associated HLH calls for further supplemented and improved in future research.

## Conclusions

In summary, our results indicate several hematology-related HLA alleles and genotypes in the Chinese population. This contributes to the elucidation of the biological functions of related hematologic diseases, thereby opening a new avenue for the development of targeted immunopathologic therapies. Ideally, these findings should be replicated in a regional distribution investigation. And prognostic indicators (age, infection, medical, and familial history) should be given greater consideration. Despite the limitations, these findings are valuable in light of bringing further insight into HLA polymorphism and clinical transplantation of the Chinese population.

## Supporting information

S1 Table HLA alleles with significant differences at each locus in AML patients compared to controls (excluding the highest-frequency alleles at each locus). (DOC)

S2 Table HLA alleles with significant differences at each locus in MDS patients compared to controls (excluding the highest-frequency alleles at each locus). (DOC)

S3 Table HLA alleles with significant differences at each locus in HLH patients compared to controls (excluding the highest-frequency alleles at each locus). (DOC)

S4 Table HLA alleles with significant differences at each locus in AA patients compared to controls (excluding the highest-frequency alleles at each locus). (DOC)

S5 Table HLA alleles with significant differences at each locus in thalassemia patients compared to controls (excluding the highest-frequency alleles at each locus). (DOC)

S6 Table HLA genotypes with significant differences at each locus in AA patients compared to controls (excluding the highest-frequency genotypes at each locus). (DOC)

S7 Table HLA genotypes with significant differences at each locus in thalassemia patients compared to controls (excluding the highest-frequency genotypes at each locus). (DOC)

S1 Dataset The minimal data set. (XLSX)
