# Supplementary material for: Investigation of HLA susceptibility alleles and genotypes with hematological disease among Chinese Han population
Source: PLoS One. 2024 Apr 9;19(4):e0281698. doi: 10.1371/journal.pone.0281698 (PMC11003630; doi:10.1371/journal.pone.0281698)
Supplement: S1 Table — (DOC) [file pone.0281698.s001.doc]

**S1 Table. HLA alleles with significant differences at each locus in AML patients compared to controls (excluding the highest-frequency alleles at each locus).**

| **HLA allele** | **Frequency in patients (%)** | **Frequency in controls (%)** | **OR (95%CI)** | **P** | **Pc** |
| --- | --- | --- | --- | --- | --- |
| **A*03:01** | 2.83 | 2.19 | 1.30 (1.15-1.48) | <0.01 | 0.02 |
| **DQB1*05:02** | 7.25 | 8.66 | 0.82 (0.76-0.89) | <0.01 | 0.02 |
