# Supplementary material for: Investigation of HLA susceptibility alleles and genotypes with hematological disease among Chinese Han population
Source: PLoS One. 2024 Apr 9;19(4):e0281698. doi: 10.1371/journal.pone.0281698 (PMC11003630; doi:10.1371/journal.pone.0281698)
Supplement: S2 Table — (DOC) [file pone.0281698.s002.doc]

**S2 Table. HLA alleles with significant differences at each locus in MDS patients compared to controls (excluding the highest-frequency alleles at each locus).**

| **HLA allele** | **Frequency in patients (%)** | **Frequency in controls (%)** | **OR (95%CI)** | **P** | **Pc** |
| --- | --- | --- | --- | --- | --- |
| **A*02:01** | 14.61 | 11.32 | 1.34 (1.21-1.49) | <0.01 | 0.01 |
| **A*02:07** | 7.09 | 9.46 | 0.73 (0.63-0.84) | <0.01 | 0.01 |
| **A*02:06** | 7.02 | 4.89 | 1.47 (1.27-1.70) | <0.01 | 0.01 |
| **A*30:01** | 6.07 | 4.58 | 1.35 (1.15-1.58) | <0.01 | 0.01 |
| **A*11:02** | 1.73 | 2.56 | 0.67 (0.50-0.89) | <0.01 | 0.03 |
| **B*13:02** | 6.28 | 4.88 | 1.31 (1.12-1.52) | <0.01 | 0.01 |
| **B*40:06** | 4.45 | 2.74 | 1.65 (1.38-1.98) | <0.01 | 0.01 |
| **B*35:01** | 4.23 | 2.65 | 1.63 (1.35-1.96) | <0.01 | 0.01 |
| **B*07:02** | 2.54 | 1.83 | 1.40 (1.10-1.77) | <0.01 | 0.04 |
| **B*15:11** | 2.47 | 1.62 | 1.53 (1.21-1.95) | <0.01 | 0.01 |
| **B*38:02** | 2.01 | 3.06 | 0.65 (0.50-0.85) | <0.01 | 0.02 |
| **C*07:02** | 14.64 | 16.59 | 0.86 (0.78-0.96) | <0.01 | 0.03 |
| **C*08:01** | 10.13 | 8.41 | 1.23 (1.08-1.39) | <0.01 | 0.02 |
| **C*06:02** | 9.35 | 7.42 | 1.29 (1.13-1.46) | <0.01 | 0.01 |
| **C*03:03** | 8.36 | 6.58 | 1.30 (1.13-1.48) | <0.01 | 0.01 |
| **C*03:02** | 5.22 | 6.54 | 0.79 (0.67-0.93) | <0.01 | 0.03 |
| **DQB1*02:01** | 3.71 | 5.37 | 0.68 (0.56-0.83) | <0.01 | 0.01 |
| **DRB1*07:01** | 8.65 | 7.34 | 1.19 (1.05-1.36) | <0.01 | 0.05 |
| **DRB1*12:02** | 7.55 | 9.02 | 0.82 (0.72-0.95) | <0.01 | 0.04 |
| **DRB1*03:01**3.78 | 5.40 | 0.69 (0.57-0.84) | <0.01 | 0.01 |  |
