# Supplementary material for: Investigation of HLA susceptibility alleles and genotypes with hematological disease among Chinese Han population
Source: PLoS One. 2024 Apr 9;19(4):e0281698. doi: 10.1371/journal.pone.0281698 (PMC11003630; doi:10.1371/journal.pone.0281698)
Supplement: S3 Table — (DOC) [file pone.0281698.s003.doc]

**S3 Table. HLA alleles with significant differences at each locus in HLH patients compared to controls (excluding the highest-frequency alleles at each locus).**

| **HLA allele** | **Frequency in patients (%)** | **Frequency in controls (%)** | **OR (95%CI)** | **P** | **Pc** |
| --- | --- | --- | --- | --- | --- |
| **B*37:01** | 3.37 | 1.14 | 3.02 (2.07-4.42) | <0.01 | 0.02 |
| **B*15:02** | 1.81 | 4.10 | 0.43 (0.26-0.72) | <0.01 | 0.02 |
