# Supplementary material for: Investigation of HLA susceptibility alleles and genotypes with hematological disease among Chinese Han population
Source: PLoS One. 2024 Apr 9;19(4):e0281698. doi: 10.1371/journal.pone.0281698 (PMC11003630; doi:10.1371/journal.pone.0281698)
Supplement: S4 Table — (DOC) [file pone.0281698.s004.doc]

**S4 Table. HLA alleles with significant differences at each locus in AA patients compared to controls (excluding the highest-frequency alleles at each locus).**

| **HLA allele** | **Frequency in patients (%)** | **Frequency in controls (%)** | **OR (95%CI)** | **P** | **Pc** |
| --- | --- | --- | --- | --- | --- |
| **A*02:01** | 15.43 | 11.32 | 1.43 (1.33-1.54) | <0.01 | <0.01 |
| **A*24:02** | 13.41 | 15.60 | 0.84 (0.78-0.90) | <0.01 | <0.01 |
| **A*02:06** | 9.21 | 4.89 | 1.97 (1.80-2.16) | <0.01 | <0.01 |
| **A*02:07** | 8.40 | 9.46 | 0.88 (0.80-0.96) | <0.01 | 0.02 |
| **A*30:01** | 6.29 | 4.58 | 1.40 (1.26-1.56) | <0.01 | <0.01 |
| **A*33:03** | 5.28 | 8.23 | 0.62 (0.55-0.70) | <0.01 | <0.01 |
| **A*02:03** | 2.70 | 4.45 | 0.60 (0.51-0.70) | <0.01 | <0.01 |
| **A*02:10** | 0.52 | 0.30 | 1.73 (1.19-2.50) | <0.01 | 0.02 |
| **A*33:01** | 0.26 | 0.12 | 2.19 (1.30-3.71) | <0.01 | 0.02 |
| **B*13:01** | 7.34 | 5.76 | 1.30 (1.17-1.43) | <0.01 | <0.01 |
| **B*13:02** | 6.54 | 4.88 | 1.36 (1.23-1.52) | <0.01 | <0.01 |
| **B*40:06** | 4.62 | 2.74 | 1.72 (1.52-1.95) | <0.01 | <0.01 |
| **B*40:02** | 4.36 | 2.09 | 2.13 (1.87-2.43) | <0.01 | <0.01 |
| **B*58:01** | 4.18 | 6.56 | 0.62 (0.55-0.71) | <0.01 | <0.01 |
| **B*48:01** | 3.59 | 2.10 | 1.74 (1.51-2.01) | <0.01 | <0.01 |
| **B*35:01** | 3.14 | 2.65 | 1.19 (1.02-1.39) | 0.02 | 0.04 |
| **B*15:02** | 2.95 | 4.10 | 0.71 (0.61-0.83) | <0.01 | <0.01 |
| **B*15:11** | 2.44 | 1.62 | 1.52 (1.28-1.80) | <0.01 | <0.01 |
| **B*07:02** | 2.32 | 1.83 | 1.27 (1.07-1.52) | <0.01 | 0.02 |
| **B*38:02** | 1.73 | 3.06 | 0.56 (0.46-0.68) | <0.01 | <0.01 |
| **B*44:03** | 1.45 | 1.90 | 0.76 (0.61-0.94) | 0.01 | 0.03 |
| **C*03:04** | 12.2 | 10.91 | 1.14 (1.05-1.23) | <0.01 | <0.01 |
| **C*08:01** | 9.69 | 8.41 | 1.17 (1.07-1.28) | <0.01 | <0.01 |
| **C*06:02** | 8.79 | 7.42 | 1.20 (1.09-1.32) | <0.01 | <0.01 |
| **C*03:03** | 7.98 | 6.58 | 1.23 (1.12-1.36) | <0.01 | <0.01 |
| **C*04:01** | 4.24 | 5.12 | 0.82 (0.72-0.93) | <0.01 | 0.01 |
| **C*03:02** | 4.11 | 6.54 | 0.61 (0.54-0.70) | <0.01 | <0.01 |
| **C*08:22** | 1.76 | 0.82 | 2.17 (1.77-2.67) | <0.01 | <0.01 |
| **C*12:03** | 1.19 | 1.70 | 0.69 (0.54-0.88) | <0.01 | 0.01 |
| **C*08:03** | 1.03 | 0.62 | 1.67 (1.28-2.17) | <0.01 | <0.01 |
| **C*04:03** | 0.58 | 1.09 | 0.53 (0.37-0.74) | <0.01 | <0.01 |
| **C*08:02** | 0.44 | 0.23 | 1.87 (1.24-2.80) | <0.01 | 0.01 |
| **DQB1*03:01** | 19.04 | 20.74 | 0.90 (0.84-0.96) | <0.01 | <0.01 |
| **DQB1*06:02** | 11.65 | 7.08 | 1.73 (1.59-1.88) | <0.01 | <0.01 |
| **DQB1*02:02** | 7.11 | 6.28 | 1.14 (1.03-1.27) | 0.01 | 0.03 |
| **DQB1*05:02** | 6.22 | 8.66 | 0.70 (0.63-0.78) | <0.01 | <0.01 |
| **DQB1*03:02** | 3.70 | 5.94 | 0.61 (0.53-0.70) | <0.01 | <0.01 |
| **DQB1*05:01** | 3.19 | 4.17 | 0.76 (0.65-0.88) | <0.01 | <0.01 |
| **DQB1*02:01** | 3.05 | 5.37 | 0.56 (0.48-0.65) | <0.01 | <0.01 |
| **DQB1*06:09** | 1.20 | 1.62 | 0.74 (0.58-0.94) | 0.01 | 0.03 |
| **DQB1*06:03** | 0.58 | 1.13 | 0.51 (0.36-0.71) | <0.01 | <0.01 |
| **DRB1*15:01** | 15.55 | 11.53 | 1.41 (1.31-1.52) | <0.01 | <0.01 |
| **DRB1*07:01** | 8.14 | 7.34 | 1.12 (1.02-1.23) | 0.02 | 0.05 |
| **DRB1*12:02** | 7.69 | 9.02 | 0.84 (0.76-0.93) | <0.01 | <0.01 |
| **DRB1*03:01** | 3.02 | 5.40 | 0.55 (0.47-0.64) | <0.01 | <0.01 |
| **DRB1*14:54** | 2.89 | 3.56 | 0.81 (0.69-0.94) | <0.01 | 0.02 |
| **DRB1*16:02** | 2.55 | 3.85 | 0.65 (0.55-0.77) | <0.01 | <0.01 |
| **DRB1*13:02** | 2.07 | 2.69 | 0.77 (0.64-0.92) | <0.01 | 0.02 |
| **DRB1*04:06** | 1.71 | 2.68 | 0.63 (0.52-0.77) | <0.01 | <0.01 |
| **DRB1*10:01** | 1.03 | 1.46 | 0.70 (0.54-0.91) | 0.01 | 0.03 |
| **DRB1*04:03** | 0.84 | 1.69 | 0.49 (0.37-0.65) | <0.01 | <0.01 |
| **DRB1*13:01** | 0.59 | 1.12 | 0.52 (0.37-0.74) | <0.01 | <0.01 |
| **DRB1*04:04** | 0.33 | 0.70 | 0.47 (0.30-0.74) | <0.01 | <0.01 |
