# Supplementary material for: Investigation of HLA susceptibility alleles and genotypes with hematological disease among Chinese Han population
Source: PLoS One. 2024 Apr 9;19(4):e0281698. doi: 10.1371/journal.pone.0281698 (PMC11003630; doi:10.1371/journal.pone.0281698)
Supplement: S5 Table — (DOC) [file pone.0281698.s005.doc]

**S5 Table. HLA alleles with significant differences at each locus in thalassemia patients compared to controls (excluding the highest-frequency alleles at each locus).**

| **HLA allele** | **Frequency in patients (%)** | **Frequency in controls (%)** | **OR (95%CI)** | **P** | **Pc** |
| --- | --- | --- | --- | --- | --- |
| **A*02:07** | 14.58 | 9.46 | 1.63 (1.38-1.94) | <0.01 | <0.01 |
| **A*02:03** | 10.19 | 4.45 | 2.44 (2.00-2.98) | <0.01 | <0.01 |
| **A*02:01** | 4.86 | 11.32 | 0.40 (0.30-0.53) | <0.01 | <0.01 |
| **A*02:06** | 3.18 | 4.89 | 0.64 (0.45-0.90) | <0.01 | 0.02 |
| **A*31:01** | 1.59 | 2.87 | 0.55 (0.34-0.88) | 0.01 | 0.03 |
| **A*30:01** | 1.31 | 4.58 | 0.28 (0.16-0.47) | <0.01 | <0.01 |
| **A*03:01** | 0.84 | 2.19 | 0.38 (0.20-0.73) | <0.01 | 0.01 |
| **A*01:01** | 0.75 | 2.68 | 0.27 (0.14-0.55) | <0.01 | <0.01 |
| **B*13:01** | 10.09 | 5.76 | 1.84 (1.50-2.24) | <0.01 | <0.01 |
| **B*58:01** | 9.53 | 6.56 | 1.50 (1.22-1.84) | <0.01 | <0.01 |
| **B*15:02** | 9.07 | 4.10 | 2.33 (1.89-2.88) | <0.01 | <0.01 |
| **B*38:02** | 4.39 | 3.06 | 1.45 (1.08-1.95) | 0.01 | 0.03 |
| **B*51:01** | 3.08 | 5.39 | 0.56 (0.39-0.79) | <0.01 | <0.01 |
| **B*15:01** | 2.34 | 4.39 | 0.52 (0.35-0.77) | <0.01 | 0.01 |
| **B*35:01** | 1.40 | 2.65 | 0.52 (0.31-0.87) | 0.01 | 0.03 |
| **B*13:02** | 1.21 | 4.88 | 0.24 (0.14-0.41) | <0.01 | <0.01 |
| **B*48:01** | 0.93 | 2.10 | 0.44 (0.24-0.82) | <0.01 | 0.02 |
| **B*07:02** | 0.75 | 1.83 | 0.40 (0.20-0.81) | 0.01 | 0.03 |
| **B*40:06** | 0.75 | 2.74 | 0.27 (0.13-0.54) | <0.01 | <0.01 |
| **B*15:11** | 0.47 | 1.62 | 0.28 (0.12-0.69) | <0.01 | 0.01 |
| **C*03:04** | 15.42 | 10.91 | 1.49 (1.26-1.76) | <0.01 | <0.01 |
| **C*08:01** | 10.93 | 8.41 | 1.34 (1.10-1.62) | <0.01 | <0.01 |
| **C*03:02** | 9.16 | 6.54 | 1.44 (1.17-1.78) | <0.01 | <0.01 |
| **C*03:03** | 4.21 | 6.58 | 0.62 (0.46-0.84) | <0.01 | <0.01 |
| **C*04:01** | 3.27 | 5.12 | 0.63 (0.45-0.88) | <0.01 | 0.01 |
| **C*04:03** | 2.34 | 1.09 | 2.18 (1.46-3.25) | <0.01 | <0.01 |
| **C*06:02** | 1.87 | 7.42 | 0.24 (0.15-0.37) | <0.01 | <0.01 |
| **DQB1*03:01** | 17.29 | 20.74 | 0.80 (0.68-0.94) | <0.01 | 0.01 |
| **DQB1*06:01** | 13.83 | 10.74 | 1.33 (1.12-1.59) | <0.01 | <0.01 |
| **DQB1*02:01** | 7.48 | 5.37 | 1.43 (1.13-1.79) | <0.01 | 0.01 |
| **DQB1*06:02** | 3.46 | 7.08 | 0.47 (0.34-0.65) | <0.01 | <0.01 |
| **DQB1*02:02** | 2.15 | 6.28 | 0.33 (0.22-0.50) | <0.01 | <0.01 |
| **DRB1*16:02** | 9.07 | 3.85 | 2.49 (2.02-3.07) | <0.01 | <0.01 |
| **DRB1*03:01** | 7.66 | 5.40 | 1.45 (1.16-1.82) | <0.01 | <0.01 |
| **DRB1*15:02** | 5.51 | 3.13 | 1.81 (1.39-2.35) | <0.01 | <0.01 |
| **DRB1*07:01** | 2.24 | 7.34 | 0.29 (0.19-0.43) | <0.01 | <0.01 |
| **DRB1*13:12** | 1.68 | 0.79 | 2.16 (1.35-3.45) | <0.01 | <0.01 |
| **DRB1*13:02** | 1.12 | 2.69 | 0.41 (0.23-0.73) | <0.01 | <0.01 |
| **DRB1*01:01** | 0.65 | 1.59 | 0.41 (0.19-0.86) | 0.02 | 0.04 |
