# Supplementary material for: Investigation of HLA susceptibility alleles and genotypes with hematological disease among Chinese Han population
Source: PLoS One. 2024 Apr 9;19(4):e0281698. doi: 10.1371/journal.pone.0281698 (PMC11003630; doi:10.1371/journal.pone.0281698)
Supplement: S6 Table — (DOC) [file pone.0281698.s006.doc]

**S6 Table. HLA genotypes with significant differences at each locus in AA patients compared to controls (excluding the highest-frequency genotypes at each locus).**

| **HLA genotype** | **Frequency in patients (%)** | **Frequency in controls (%)** | **OR (95%CI)** | **P** | **Pc** |
| --- | --- | --- | --- | --- | --- |
| **A*02:01-A*24:02** | 4.43 | 3.54 | 1.26 (1.05-1.51) | 0.01 | 0.03 |
| **A*02:06-A*11:01** | 3.63 | 2.06 | 1.79 (1.46-2.19) | <0.01 | <0.01 |
| **A*02:01-A*02:06** | 2.96 | 1.25 | 2.41 (1.93-3.02) | <0.01 | <0.01 |
| **A*02:06-A*24:02** | 2.58 | 1.53 | 1.70 (1.34-2.16) | <0.01 | <0.01 |
| **A*02:01-A*02:01** | 2.48 | 1.48 | 1.69 (1.33-2.15) | <0.01 | <0.01 |
| **A*02:01-A*30:01** | 2.41 | 1.28 | 1.90 (1.48-2.43) | <0.01 | <0.01 |
| **A*24:02-A*30:01** | 2.23 | 1.42 | 1.58 (1.23-2.04) | <0.01 | <0.01 |
| **A*02:07-A*24:02** | 2.09 | 2.96 | 0.70 (0.54-0.91) | <0.01 | 0.03 |
| **A*11:01-A*33:03** | 2.02 | 4.01 | 0.49 (0.38-0.64) | <0.01 | <0.01 |
| **A*24:02-A*33:03** | 1.32 | 2.45 | 0.54 (0.39-0.74) | <0.01 | <0.01 |
| **A*02:03-A*11:01** | 1.29 | 2.37 | 0.54 (0.39-0.75) | <0.01 | <0.01 |
| **A*02:07-A*33:03** | 0.70 | 1.59 | 0.43 (0.28-0.68) | <0.01 | <0.01 |
| **A*02:03-A*24:02** | 0.63 | 1.38 | 0.45 (0.28-0.72) | <0.01 | <0.01 |
| **A*02:03-A*02:07** | 0.28 | 1.04 | 0.27 (0.13-0.54) | <0.01 | <0.01 |
| **B*40:01-B*46:01** | 2.02 | 3.21 | 0.62 (0.48-0.81) | <0.01 | 0.01 |
| **B*40:01-B*40:01** | 1.01 | 1.75 | 0.57 (0.40-0.83) | <0.01 | 0.01 |
| **B*40:01-B*58:01** | 1.01 | 1.77 | 0.57 (0.39-0.82) | <0.01 | 0.01 |
| **B*46:01-B*58:01** | 0.94 | 1.64 | 0.57 (0.39-0.83) | <0.01 | 0.01 |
| **B*40:01-B*40:06** | 0.91 | 0.52 | 1.76 (1.18-2.62) | <0.01 | 0.02 |
| **B*38:02-B*46:01** | 0.35 | 0.81 | 0.43 (0.23-0.80) | <0.01 | 0.03 |
| **B*38:02-B*40:01** | 0.31 | 0.77 | 0.41 (0.21-0.79) | <0.01 | 0.01 |
| **B*15:02-B*58:01** | 0.21 | 0.62 | 0.34 (0.15-0.75) | <0.01 | 0.02 |
| **C*01:02-C*01:02** | 3.91 | 3.10 | 1.27 (1.05-1.54) | 0.01 | 0.04 |
| **C*03:03-C*03:04** | 1.99 | 1.28 | 1.56 (1.19-2.05) | <0.01 | <0.01 |
| **C*06:02-C*08:01** | 1.99 | 1.19 | 1.68 (1.29-2.21) | <0.01 | <0.01 |
| **C*03:04-C*06:02** | 1.95 | 1.38 | 1.42 (1.09-1.87) | 0.01 | 0.04 |
| **C*03:03-C*06:02** | 1.74 | 1.12 | 1.56 (1.17-2.08) | <0.01 | 0.01 |
| **C*01:02-C*03:02** | 1.50 | 2.29 | 0.65 (0.48-0.88) | <0.01 | 0.02 |
| **C*03:02-C*07:02** | 1.36 | 2.28 | 0.59 (0.43-0.81) | <0.01 | <0.01 |
| **DQB1*03:03-DQB1*03:03** | 5.93 | 2.89 | 2.12 (1.80-2.49) | <0.01 | <0.01 |
| **DQB1*03:01-DQB1*06:02** | 5.75 | 2.92 | 2.03 (1.72-2.38) | <0.01 | <0.01 |
| **DQB1*03:03-DQB1*06:02** | 4.81 | 2.26 | 2.19 (1.83-2.61) | <0.01 | <0.01 |
| **DQB1*03:01-DQB1*06:01** | 3.00 | 4.40 | 0.67 (0.54-0.83) | <0.01 | <0.01 |
| **DQB1*02:02-DQB1*06:02** | 2.27 | 1.22 | 1.88 (1.46-2.43) | <0.01 | <0.01 |
| **DQB1*03:01-DQB1*05:02** | 2.23 | 3.41 | 0.65 (0.50-0.83) | <0.01 | <0.01 |
| **DQB1*03:01-DQB1*03:02** | 1.36 | 2.42 | 0.56 (0.40-0.77) | <0.01 | <0.01 |
| **DQB1*02:01-DQB1*03:01** | 0.98 | 2.25 | 0.43 (0.29-0.62) | <0.01 | <0.01 |
| **DQB1*02:01-DQB1*06:01** | 0.63 | 1.25 | 0.50 (0.31-0.80) | <0.01 | 0.01 |
| **DQB1*03:02-DQB1*06:01** | 0.59 | 1.31 | 0.45 (0.28-0.72) | <0.01 | <0.01 |
| **DRB1*09:01-DRB1*09:01** | 5.72 | 2.52 | 2.35 (1.99-2.77) | <0.01 | <0.01 |
| **DRB1*08:03-DRB1*09:01** | 5.51 | 2.10 | 2.72 (2.30-3.21) | <0.01 | <0.01 |
| **DRB1*07:01-DRB1*15:01** | 3.14 | 1.81 | 1.76 (1.42-2.18) | <0.01 | <0.01 |
| **DRB1*11:01-DRB1*15:01** | 2.37 | 1.29 | 1.86 (1.45-2.39) | <0.01 | <0.01 |
| **DRB1*09:01-DRB1*15:02** | 2.16 | 0.91 | 2.41 (1.85-3.13) | <0.01 | <0.01 |
| **DRB1*03:01-DRB1*09:01** | 1.01 | 1.61 | 0.62 (0.43-0.90) | 0.01 | 0.04 |
| **DRB1*08:03-DRB1*12:02** | 0.49 | 1.28 | 0.38 (0.22-0.64) | <0.01 | <0.01 |
| **DRB1*03:01-DRB1*12:02** | 0.42 | 0.97 | 0.43 (0.24-0.76) | <0.01 | 0.01 |
