# Supplementary material for: Investigation of HLA susceptibility alleles and genotypes with hematological disease among Chinese Han population
Source: PLoS One. 2024 Apr 9;19(4):e0281698. doi: 10.1371/journal.pone.0281698 (PMC11003630; doi:10.1371/journal.pone.0281698)
Supplement: S7 Table — (DOC) [file pone.0281698.s007.doc]

**S7 Table. HLA genotypes with significant differences at each locus in thalassemia patients compared to controls (excluding the highest-frequency genotypes at each locus).**

| **HLA genotype** | **Frequency in patients (%)** | **Frequency in controls (%)** | **OR (95%CI)** | **P** | **Pc** |
| --- | --- | --- | --- | --- | --- |
| **A*02:07-A*11:01** | 9.53 | 4.92 | 2.04 (1.52-2.72) | <0.01 | <0.01 |
| **A*02:03-A*11:01** | 5.42 | 2.37 | 2.36 (1.62-3.44) | <0.01 | <0.01 |
| **A*02:07-A*24:02** | 5.23 | 2.96 | 1.81 (1.23-2.65) | <0.01 | 0.02 |
| **A*02:03-A*02:07** | 4.30 | 1.04 | 4.29 (2.81-6.55) | <0.01 | <0.01 |
| **A*02:01-A*24:02** | 1.31 | 3.54 | 0.36 (0.17-0.76) | <0.01 | 0.03 |
| **B*13:01-B*46:01** | 3.74 | 1.42 | 2.70 (1.72-4.23) | <0.01 | <0.01 |
| **B*15:02-B*46:01** | 3.55 | 1.14 | 3.20 (2.02-5.09) | <0.01 | <0.01 |
| **B*13:01-B*40:01** | 2.99 | 1.47 | 2.06 (1.25-3.40) | <0.01 | 0.03 |
| **B*13:01-B*15:02** | 2.80 | 0.59 | 4.88 (2.90-8.22) | <0.01 | <0.01 |
| **B*15:02-B*40:01** | 2.62 | 1.12 | 2.37 (1.39-4.05) | <0.01 | 0.03 |
| **C*03:04-C*08:01** | 3.93 | 1.82 | 2.20 (1.42-3.42) | <0.01 | <0.01 |
| **DQB1*05:02-DQB1*05:02** | 7.10 | 1.21 | 6.24 (4.46-8.74) | <0.01 | <0.01 |
| **DQB1*05:02-DQB1*06:01** | 6.54 | 1.95 | 3.51 (2.49-4.97) | <0.01 | <0.01 |
| **DQB1*03:03-DQB1*05:02** | 5.23 | 2.61 | 2.06 (1.41-3.03) | <0.01 | <0.01 |
| **DQB1*02:01-DQB1*06:01** | 2.80 | 1.25 | 2.28 (1.36-3.83) | <0.01 | 0.03 |
| **DRB1*03:01-DRB1*15:01** | 2.80 | 1.23 | 2.32 (1.38-3.89) | <0.01 | 0.03 |
| **DRB1*15:01-DRB1*16:02** | 2.62 | 0.92 | 2.91 (1.70-4.97) | <0.01 | <0.01 |
| **DRB1*09:01-DRB1*14:54** | 2.43 | 1.12 | 2.20 (1.26-3.82) | <0.01 | 0.03 |
